# The Outcomes of XEN Gel Stent Implantation: A Systematic Review and Meta-Analysis

**DOI:** 10.3389/fmed.2022.804847

**Published:** 2022-02-04

**Authors:** Xuan-zhu Chen, Zhi-qiao Liang, Kang-yi Yang, Kun Lv, Yao Ma, Meng-yang Li, Hui-juan Wu

**Affiliations:** Beijing Key Laboratory of Diagnosis and Therapy of Retinal and Choroid Diseases, Department of Ophthalmology, College of Optometry, Eye Diseases and Optometry Institute, Peking University People's Hospital, Peking University Health Science Center, Beijing, China

**Keywords:** XEN gel stent, meta-analysis, minimally invasive glaucoma surgeries, trabeculectomy, complications

## Abstract

**Purpose:**

XEN gel stents are used for the treatment of open-angle glaucoma (OAG), including primary and secondary glaucoma that are uncontrolled by previous medical therapy and cases with previous failed surgery. Our aim was to systematically review of the clinical data of currently published ab-interno XEN gel stents with an emphasis on intraocular pressure (IOP), antiglaucoma medication outcomes, and safety profiles.

**Methods:**

We analyzed all of the publications (MEDLINE, EMBASE, Cochrane Library) on the ab-interno XEN gel stent to evaluate the reduction in IOP and antiglaucoma medications following the procedure. The primary outcomes measured for the meta-analysis were reduction in IOP and anti-glaucoma medications. The secondary outcome were adverse events. For each study, we used a random effects analysis model to calculate the mean difference and 95% confidence intervals for the continuous results (reduction in IOP and antiglaucoma medications) using the inverse variance statistical method.

**Results:**

Five hundred twenty-seven articles were checked and 56 studies were found to be relevant with a total of 4,410 eyes. There was a significant reduction in IOP as well as in the number of medications required in patients treated with ab-interno XEN implant either alone or combined with cataract surgery. This new treatment for various types of glaucoma reduced the IOP by 35% to a final average close to 15 mmHg. This reduction was accompanied by a decrease in the number of antiglaucoma medications in all the studies, approximately 2 classes of medication at the price of more needlings. The overall complete success rate was 21.0–70.8% after 2 years using strict criteria originally designed to record success rate in filtration surgery. The incidence of complications vision-threatening was low at <1%.

**Conclusions:**

XEN gel stent was effective and safe for primary and secondary OAG. Further studies should be performed to investigate the impact of ethnicity on the success and failure rate after XEN implantation.

## Introduction

Minimally invasive glaucoma surgeries (MIGSs) are surgical interventions for mild or moderate glaucoma via the ab-interno or ab-externo approach for lowering intraocular pressure (IOP) with minimal or no scleral dissection, aiming to provide a safe profile and rapid recovery compared with traditional surgery (1, 2). MIGSs always target Schlemm's canal and the suprachoroidal space to lower IOP, which is the main complement of outflow resistance in the pathophysiology of glaucoma, whereas the XEN gel stent is the first MIGS procedure to drain aqueous to subconjunctival space. It is a 6-mm hydrophilic tube of a collagen-derived gelatin cross-linked with glutaraldehyde to prevent degradation in the tissue given the lack of a foreign-body reaction (3). XEN gel stents are preloaded in a specifically designed handheld inserter, and there are three models with different inner diameters of 140, 63, and 45 μm, which were chosen to reduce the occurrence of postoperative hypotony by the flow resistance of the tube itself according to the Hagen–Poiseuille equation (4). The outflow resistance was 0–1, 2–3, and 6–8 mmHg for devices with inner diameters of 140, 63, and 45 μm, respectively (5–7). To exclude the difference in outcomes caused by different inner diameters, the meta-analysis only included the studies focused on the devices with inner diameter of 45 μm.

XEN gel stents are used for the treatment of open-angle glaucoma, including primary and secondary glaucoma that are uncontrolled by previous medical therapy and cases with previous failed surgery (8–10). Lewczuk et al. (8) demonstrated that repeat XEN implantation might be beneficial for patients previously undergone multiple glaucoma surgeries. However, the surgical success rate after XEN implantation did not differ from that in patients with previous anti-glaucoma surgeries. Meanwhile, Lewczuk et al. (11) have demonstrated that the applied of XEN surgery appears to show promising results in patients with uncontrolled glaucoma. Patients with Shaffer 3 or 4 angles were considered as a contraindication because the iris may cause occlusion of the anterior chamber (AC) portion of the XEN implant; patients with Shaffer 2 or less could be selected provided combined with the lens extraction. Of all published studies that reported glaucoma subtypes, primary open-angle glaucoma (POAG) accounts for greater than three-fourths (75.8%). The second largest subgroup was pseudoexfoliation glaucoma in total (13.6%). Other types of glaucoma include pigmentary glaucoma, uveitic secondary glaucoma, juvenile open-angle glaucoma, and steroid-induced glaucoma, etc. Some studies enrolled patients with ocular hypertension to reduce IOP (12–16). Other studies introduced XEN to patients with primary angle-closure glaucoma (PACG), although it was originally a contraindication (9, 13, 16–21). Details on the degree of narrow angles were not reported in all studies. Sng et al. (20) reported no significant difference in the IOP reduction (*p* = 0.503) or in the decrease in the number of antiglaucoma medications (*p* = 0.332) between eyes with POAG and PACG at 12 months after XEN implantation.

XEN gel stents obtained the CE mark in December 2015 and were approved by the Food and Drug Administration (FDA) in November 2016. Since then, many studies have been published. However, no randomized control trials (RCTs) have not been performed to date. Our aim was to systematically review of the clinical data of currently published ab-interno XEN gel stents with an emphasis on IOP, antiglaucoma medication outcomes, and safety profiles.

## Materials and Methods

### Search Strategy

This meta-analysis is reported on the basis of the Preferred Reporting Items for Systematic Reviews and Meta-Analyses (PRISMA) Statement (22). Two researchers independently selected relevant studies by searching the PubMed database, the Cochrane Library, and EMBASE using the MeSH terms, including “glaucoma,” “open-angle,” “XEN,” “micro stent,” and “gel implant.” We also conducted a manual search using references of major articles published in English. The studies were published between September 14, 2015 and December 15, 2021.

### Study Selection and Data Extraction

The inclusion criteria were as follows: (1) prospective or retrospective case series or cohort; (2) glaucoma patients without restriction for age, sex, ethnicity, use of antiglaucoma medications; (3) XEN implantation combined with phacoemulsification or not; and (4) IOP, antiglaucoma medications, success rate, failure rate, reoperation rate, and complications. Only the studies with the longest follow-up were included for studies with overlapping populations. Case reports and reviews with < 12 months of follow-up and articles lacking essential information for meta-analysis were excluded.

The following data were independently extracted from published studies by two researchers (X-zC and Z-qL) using standardized protocols: first author's last name, year of publication, study design, number of eyes enrolled, the number of different glaucoma subtypes, differences in surgical technique between studies, success, failure and reoperation rate, and complications during follow-up.

### Outcome Measures

The primary outcomes measured for the meta-analysis were reduction in IOP and anti-glaucoma medications. The secondary outcome were adverse events.

### Statistical Analysis

Data were processed using REVMAN (Version 5.0; The Cochrane Collaboration, Copenhagen, Denmark). For each study, we used a random effects analysis model to calculate the mean difference (MD) and 95% confidence intervals (CIs) for the continuous results (change in IOP and antiglaucoma medications) using the inverse variance statistical method.

The between-study heterogeneity was tested by the chi-square-based Cochran's statistics and the inconsistency index (I-squared value) (23). *I*^2^ testing with values >50% indicated moderate-to-high heterogeneity. *P* < 0.05 was considered statistically significant. Given the limited number of trials involved in the final analysis, we did not perform subgroup analysis and asymmetry assessment of the funnel plot for evaluating publication bias.

### Surgical Technique

The surgical techniques of most studies followed similar key steps. Briefly, the preloaded injector was inserted into viscoelastic gel-filled AC through a corneal paracentesis incision. The implant entered into the subconjunctival space to a distance of 3 mm from the limbus without a conjunctival peritomy. For patients with indications for cataract surgery, the standard phacoemulsification technique was used. After the operation, as mentioned above, XEN was implanted.

The possible sources of variation in technique include the inner diameter of the gel stent and whether using mitomycin C (MMC) was used before implantation. Most studies used gel stents with an inner diameter of 45 μm, and a few studies used gel stents with lumen diameters of 140 μm (7) and 63 μm (24–26). To exclude the difference in outcomes caused by different inner diameters, the meta-analysis only included the studies focused on the devices with inner diameter of 45 μm. MMC was introduced as an adjunctive agent in the area where the XEN gel stent was to be implanted in most studies. In the studies (7, 26) that did not use intraoperative MMC, no additional bleb-related complications were reported.

In most studies, all antiglaucoma medications were stopped on the day of surgery. In addition, 1% prednisolone acetate drops were placed in the operative eye followed by a slow taper over. Prophylactic antibiotic drops were continued according to the patients' condition. When the target IOP was not reached during follow-up postoperatively, or when the progression of glaucoma was found, further treatment was performed, including reintroduction of IOP-lowering medications, needling revision, XEN replacement, and alternative filtering surgery or glaucoma drainage device surgery in refractory cases.

## Results

### Literature Search

**Figure 1:**
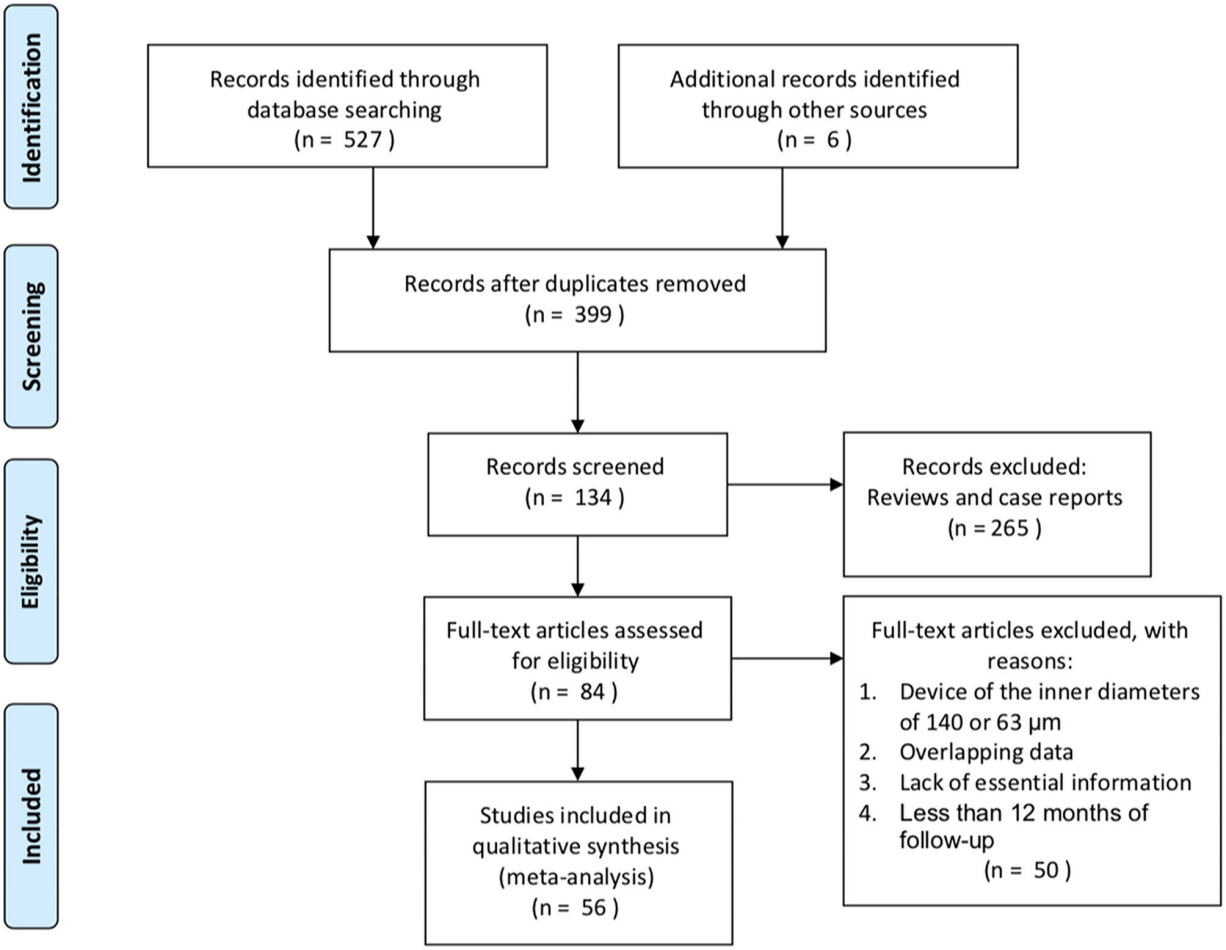
Flow chart of included studies for the meta-analysis.

We identified 527 potentially eligible literature citations, of which 56 were included in this meta-analysis with a total of 4,410 eyes. The aim of most studies was to determine the postoperative course after XEN implantation in Caucasian patients with glaucoma. However, a few studies focused on Asian patients (9, 20, 27–29) and black or Afro-Latino patients (30, 31) with glaucoma. The flow chart of the search results is shown in Figure 1.

### Overall Results

Table 1 provides the detailed characteristics of the participants from the 56 studies. There were no RCTs involving XEN. These studies were published between September 14, 2015 and December 15, 2021. The main participants in most studies were patients with POAG. However, some studies focused on the efficacy of XEN implantation in the treatment of secondary open-angle glaucoma, including pseudoexfoliative glaucoma (52, 60, 67–70) and glaucoma secondary to uveitis (34). Furthermore, a few studies introduced this gel stent to narrow- or close-angle glaucoma (13, 15, 16, 18–21, 50). All patients were treated and followed as routine clinical practice between May 2013 and February 2020. The mean sample size was 79 ± 67. The average follow-up time was 17.0 ± 8.1 months, and the follow-up loss rate of most studies was reported as <20%.

**Table 1:** Intraocular pressure (IOP) and medication outcomes following XEN implantation.

**References**	**Year**	**Type of study**	**Total**	**Length of study (months)**	**(Baseline IOP (mmHg) mean ±SD)**	**(Final IOP (mmHg) an ±SD)**	**IOP decrease (%)**	**Mean decrease in medication**
**Standalone XEN outcomes**								
Lenzhofer et al. (26)	2019	Prospective	69	24	22.5 ± 6.5	13.0 ± 5.2	42.2	2.2
Gillmann et al. (17)	2020	Prospective	26	36	21.0 ± 7.4	12.9 ± 2.9	38.6	2.1
Ozal et al. (32)	2017	Retrospective	9	12	36.7 ± 4.1	17.0 ± 4.2	53.7	3.1
Reitsamer et al. (33)	2019	Prospective	106	24	21.7 ± 3.8	15.4 ± 4.2	29.0	1.5
Qureshi et al. (34)	2019	Retrospective	37	12	36.1 ± 9.6	12.6 ± 4.1	65.1	3.1
Post et al. (35)	2020	Not reported	20	12	21.6 ± 2.3	17.7 ± 2.1	18.1	1.6
Bravetti et al. (27)	2020	Retrospective	60	12	29.9 ± 13.3	15.2 ± 6.6	49.2	1.7
Kalina et al. (36)	2019	Prospective	20	12	24.2 ± 8.2	13.0 ± 4.5	46.3	Not reported
Scheres et al. (37)	2020	Retrospective	41	24	19.2 ± 4.4	13.8 ± 3.8	28.1	1.6
Chao et al. (28)	2020	Retrospective	37	12	21.7 ± 7.7	15.0 ± 2.0	30.9	2.1
Olgun et al. (38)	2020	Retrospective	51	24	24.4 ± 4.3	14.2 ± 2.2	33.6	1.4
Marcos Parra et al. (39)	2019	Retrospective	17	12	22.2 ± 6.8	15.5^*^	30.2	2.3
Schargus et al. (14)	2020	Retrospective	153	12	23.9 ± 7.4	15.4 ± 5.1	35.6	1.8
Theilig et al. (40)	2020	Retrospective	48	12	24.4 ± 6.6	16.6 ± 5.9	32.0	1.7
Stoner et al. (15)	2021	Retrospective	52	12	21.4 ± 8.3	13.0 ± 0.6	39.3	1.3
Gillmann (17)	2021	Prospective	26	36	21.0 ± 7.4	12.9 ± 2.9	38.6	2.1
Düzgün et al. (10)	2021	Retrospective	14	12	24.1 ± 2.7	12.2 ± 2.9	49.4	2.4
Schargus (41)	2021	Retrospective	38	24	24.1 ± 4.7	15.7 ± 3.0	34.9	2.5
Nuzzi et al. (42)	2021	Retrospective	23	36	24.9 ± 6.1	19.6 ± 2.1	21.3	Not reported
Eraslan et al. (43)	2021	Retrospective	26	12	23.7 ± 6.0	16.3 ± 3.0	31.2	1.9
Bormann (44)	2021	Retrospective	31	12	23.5 ± 6.5	18.0 ± 5.3	23.4	1.7
Lewczuk et al. (8)	2021	Retrospective	43	24	25.0 ± 7.5	16.8 ± 5.1	32.8	Not reported
Wanichwecharungruang and Ratprasatporn (9)	2021	Retrospective	57	24	21.6 ± 4.0	14.6 ± 3.5	32.4	1.7
Lewczuk et al. (11)	2021	Retrospective	72	24	24.8 ± 8.0	17.5 ± 5.8	29.4	Not reported
**Combined phacoemulsification with XEN outcomes**								
Pérez-Torregrosa et al. (45)	2016	Prospective	30	12	21.2 ± 3.4	15.0 ± 2.5	29.2	2.9
Galal et al. (30)	2017	Prospective	10	12	16.0 ± 4.0	14.0 ± 3.0	12.5	1.5
Ozal et al. (32)	2017	Retrospective	6	12	35.2 ± 3.2	15.5 ± 2.3	56.0	3.5
De Gregorio et al. (46)	2018	Prospective	41	12	22.5 ± 3.7	13.1 ± 2.4	41.8	2.2
Kalina et al. (36)	2019	Prospective	27	12	21.0 ± 6.5	13.6 ± 2.9	35.2	Not reported
Lenzhofer et al. (47)	2019	Prospective	68	24	23.4 ± 6.3	12.7 ± 6.9	45.7	1.5
Reitsamer et al. (33)	2019	Prospective	79	24	21.0 ± 3.4	14.9 ± 4.5	29.0	1.5
Sng et al. (20)	2019	Prospective	31	12	22.1 ± 3.6	12.1 ± 2.6	45.2	1.3
Gillmann et al. (17)	2020	Prospective	66	36	20.0 ± 6.9	12.9 ± 3.4	35.5	1.4
Olgun et al. (38)	2020	Retrospective	45	24	24.8 ± 3.5	13.4 ± 1.4	46.0	1.6
Marcos Parra et al. (39)	2019	Retrospective	48	12	18.0 ± 4.5	14.3^*^	20.6	2
Theillac et al. (48)	2020	Retrospective	47	12	20.8 ± 6.8	16.2 ± 2.8	22.1	1.7
Theilig et al. (40)	2020	Retrospective	52	12	24.8 ± 6.9	16.4 ± 4.2	33.9	1.7
Subaşı (49)	2020	Retrospective	30	24	20.4 ± 4.8	14.8 ± 1.9	19.7	2.0
Gillmann (17)	2021	Prospective	76	36	20.0 ± 6.9	12.9 ± 3.4	35.5	1.4
Schargus (41)	2021	Retrospective	32	24	25.4 ± 5.6	14.7 ± 3.2	42.1	2.3
Eraslan et al. (43)	2021	Retrospective	32	12	24.4 ± 7.2	16.4 ± 2.3	32.8	2.1
**Both outcomes**								
Fea et al. (12)	2020	Prospective	171	12	23.9 ± 7.0	15.5 ± 3.9	35.1	2.5
Fernández-García et al. (24)	2020	Retrospective	40	12	18.0 ± 5.2	14.6 ± 1.9	18.9	0.6
Gabbay et al. (50)	2019	Retrospective	151	24	22.1 ± 6.5	14.5 ± 3.3	34.4	2.27
Grover et al. (51)	2017	Prospective	65	12	25.1 ± 3.7	15.9 ± 5.2	36.7	1.8
Heidinger et al. (18)	2019	Retrospective	199	12	22.8 ± 6.9	17.1 ± 5.9	25.0	1.1
Hengerer et al. (19)	2017	Retrospective	242	12	32.2 ± 9.1	14.2 ± 4.0	55.9	2.8
Ibáñez-Muñoz et al. (52)	2019	Retrospective	21	12	21.1 ± 3.8	15.2 ± 3.9	28.0	1.7
Karimi et al. (53)	2019	Retrospective	258	18	19.3 ± 6.0	13.5 ± 3.3	30.1	1.4
Laroche et al. (31)	2019	Retrospective	12	12	15.3 ± 6.2	12.9 ± 4.5	15.7	1.8
Mansouri et al. (29)	2018	Prospective	149	12	20.0 ± 7.1	13.9 ± 4.3	30.5	1.4
Rauchegger et al. (54)	2020	Retrospective	79	24	23.4 ± 7.9	14.8 ± 4.4	34.2	1.7
Smithet al. (13)	2019	Retrospective	68	12	22.1 ± 6.4	14.8 ± 5.1	33.0	1.8
Tan et al. (55)	2018	Retrospective	39	12	24.9 ± 7.8	14.5 ± 3.4	41.8	Not reported
Widder (56)	2018	Retrospective	233	18	24.3 ± 6.6	16.8 ± 7.6	30.9	2.4
Wagner et al. (57)	2020	Retrospective	82	12	Not reported	Not reported	28.9	Not reported
Teus et al. (58)	2019	Cross-Sectional	10	Not reported	19.5 ± 6.4	Not reported	43.6	Not reported
Tan et al. (59)	2021	Retrospective	50	12	23.5 ± 8.5	14.7 ± 0.8	22.9	Not reported
Busch et al. (60)	2020	Retrospective	113	12	23.8 ± 6.2	16.1 ± 4.7	32.4	2.1
Barão (61)	2020	Cross-Sectional	25	18	22.8 ± 8.4	18.2 ± 9.6	20.2	1.8
Ucar and Cetinkaya (62)	2020	Retrospective	44	12	27.4 ± 8.6	11.3 ± 1.7	58.8	2.4
Reitsamer et al. (63)	2021	Retrospective	76	36	20.7 ± 5.1	13.9 ± 4.3	32.9	1.4
Gabbay et al. (64)	2021	Retrospective	205	36	22.6 ± 7.0	14.0 ± 2.9	38.1	2.0
Baser (2020)	2020	Retrospective	29	36	24.5 ± 8.7	15.6 ± 3.6	36.3	2.0
José et al. (21)	2021	Retrospective	94	24	24.0 ± 5.2	13.5 ± 5.9	43.8	0.4
Nicolaou et al. (16)	2021	Retrospective	186	24	18.1 ± 5.8	12.6 ± 3.1	30.4	0.8
Olsen (65)	2021	Retrospective	27	12	17.8 ± 7.4	11.5 ± 3.3	35.4	2.2
Navero-Rodríguez et al. (66)	2020	Retrospective	39	12	Not reported	Not reported	19.7	2.2

*The trials that only reported one set of results for both standalone and combined cases defined as both outcomes*.

* *Standard deviation was not reported*.

The Tube vs. Trabeculectomy (TVT) Study (71) defined success as IOP ≤ 21 mmHg or 20% lower than baseline without reoperation. Eyes meeting the above criteria and not receiving supplemental medical therapy were considered complete successes. Eyes requiring complementary medications were defined as qualified successes. This definition was used in seven studies (13, 14, 16, 34, 50, 53, 55). The complete success rate was 74.0–89.2% after 1 year (based on *n* = 4 studies) and 21.0–70.8% after 2 years (*n* = 3). The qualified success rate was 60.0–90.2% after 1 year (*n* = 4) and 34.0–86.0% after 2 years (*n* = 3). If the absolute IOP threshold was decreased to 18 mmHg (13, 18, 19, 28, 29, 36, 43, 50, 54), the qualified success rate was 25.0–90.6% and the complete success rate was 15.4–76.7% at the last follow-up. In a 4-year follow-up study (26), which was the longest follow-up period, XEN with an inner diameter of 63 μm was applied in patients with open-angle glaucoma. Fifty-three percent of patients achieved qualified surgical success, and 25% of patients achieved complete success after 4 years.

### Decreasing IOP and Reducing Antiglaucoma Medications

**Figure 2:**
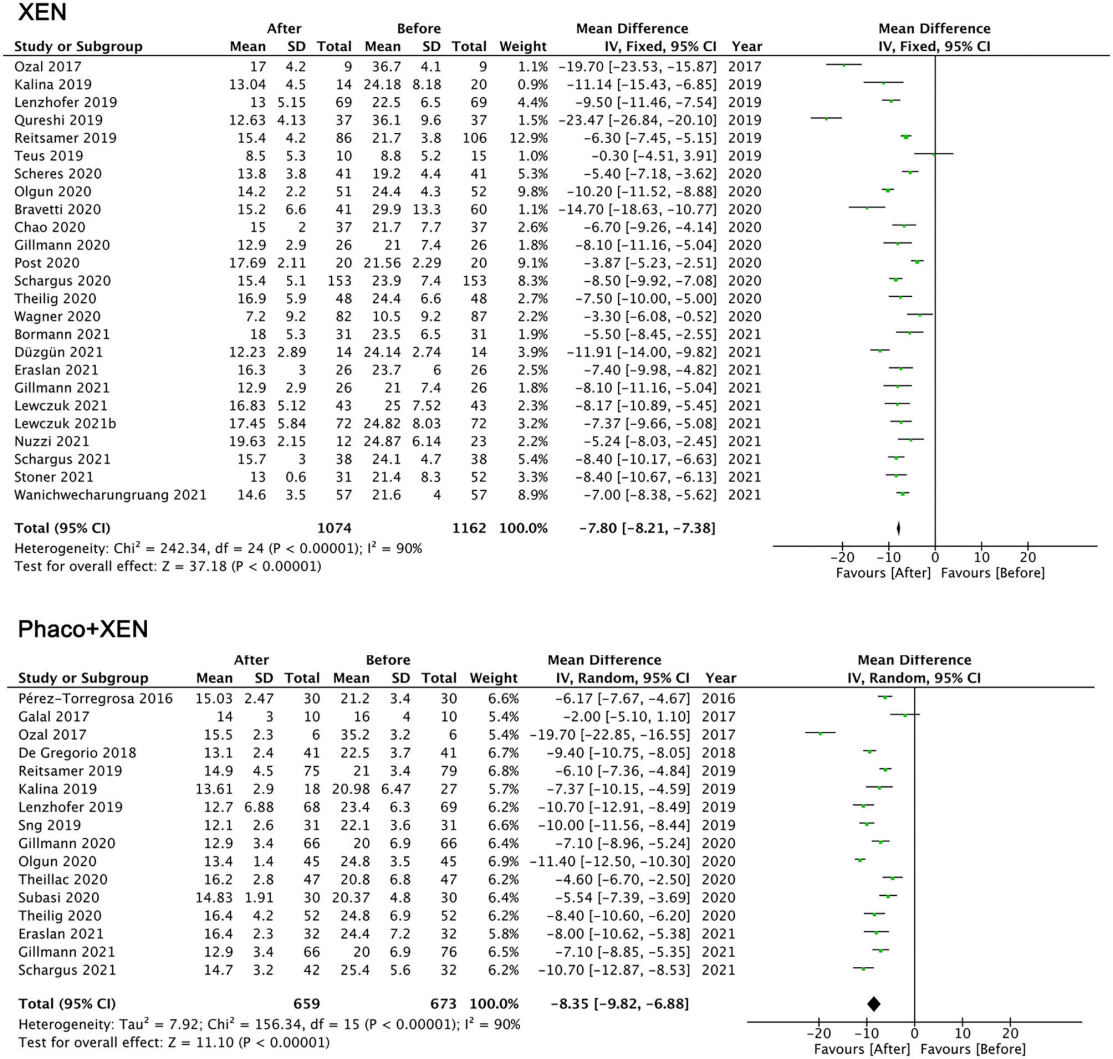
Mean difference (MD) of the reduction in intraocular pressure between the baseline and the final follow-up by XEN alone, phacoemulsification combined with XEN (phaco + XEN).

A pooled analysis with a random-effects model showed that the IOP of the final follow-up was significantly lower than that of the baseline: XEN standalone MD = −7.80 mmHg (95% CI −7.38 to −8.21, *p* < 0.001) and Phaco + XEN MD = −8.35 mmHg (95% CI −6.88 to −9.82, *p* < 0.001; Figure 2). For XEN standalone studies, patients with glaucoma secondary to uveitis had the greatest reduction in IOP [MD = −23.47 in Qureshi's study (34)] followed by studies on XEN introduced in refractory glaucoma subgroups [MD = −19.70 (32), −14.70, and −11.70 (27, 36)]. Overall, XEN lowers IOP by approximately 35% to a final average close to 15 mmHg. In most studies, the proportion of IOP decreases was >30%. Only 6 studies reported that IOP decreased by <20% (24, 30, 31, 35, 49, 66). A common feature was found among these studies, which was that the baseline IOP was at a relatively low level of <22 mmHg. Patients in 4 studies attained a >50% of decrease in IOP after XEN implantation (19, 32, 34, 62). We found that the baseline IOP in these studies was in a relatively high level and most of them were >32 mmHg.

**Figure 3:**
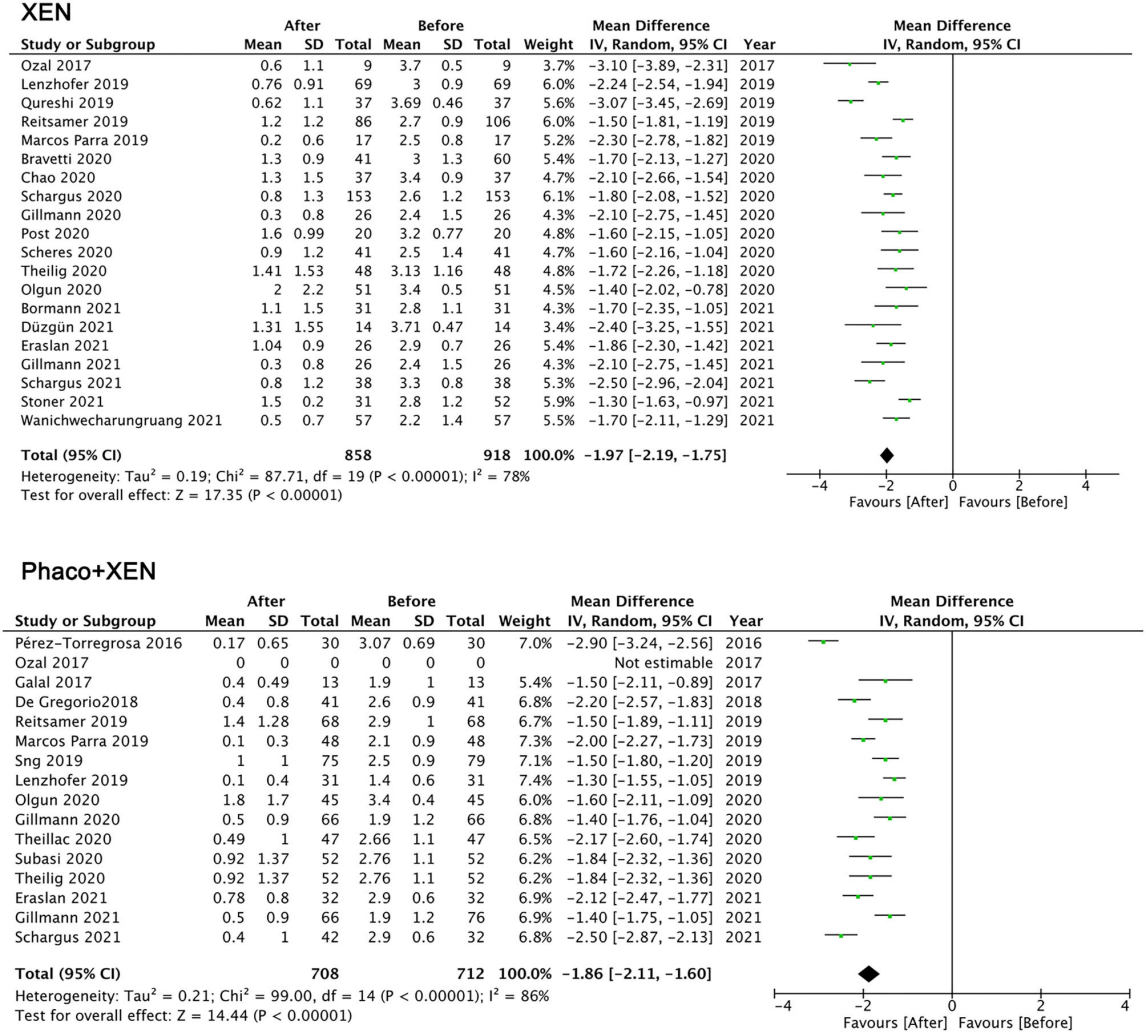
Mean difference (MD) of the reduction in anti-glaucoma medications between the baseline and the final follow-up by XEN alone, phacoemulsification combined with XEN (phaco + XEN).

The number of antiglaucoma medications also showed a significant reduction: XEN standalone MD =-1.97 (95% CI −1.75 to −2.19, *p* < 0.001) and Phaco + XEN MD = −1.86 (95% CI −1.60 to −2.11, *p* < 0.001; Figure 3). Despite high heterogeneity in all analysis (*I*^2^ > 75%), a limited meta-analysis showed that the IOP and medications for both XEN standalone and Phaco + XEN were significantly decreased. Given that this review combined studies of different sample sizes, glaucoma subtypes, follow-up durations, and races, heterogeneity can be predicted. Due to the variable research design, limited number of clinical trials, and lack of specific data of subjects, it is difficult to explore the source of heterogeneity.

### Risk Factors for Failure

Five studies analyzed the risk factors for failure from different points of view (12, 17, 20, 26, 29).

Many potential predictive factors for failure were taken into consideration, including age, ethnicity, systemic diseases, sex, glaucoma subtype, number of pre-operative antiglaucoma medications, pre-operative medicated and unmedicated IOP, a diagnosis of POAG, baseline MD and combined cataract surgery.

Three studies (12, 20, 29) demonstrated that none of the analyzed factors were statistically significantly (*P* > 0.05) associated with failure. One of the predictor of surgical failure is being male. The other two studies showed similar results: males had more failures than females [95% CI for effect: 4.3–56%, *p* = 0.023 (26); OR: 3.57, *p* = 0.030 (17)]. However, Gabbay et al. (64) reported that female was found to be 2.3 times (95% CI 1.1–4.9, *p* = 0.02) more likely to fail. Conflicting results have also been reported by Mansouri et al. (29) and Sng et al. (20). Gillmann et al. (17) postulated that a diagnosis of POAG (OR: 4.52; *p* = 0.005) and requiring needling revisions (OR: 4.56; *p* = 0.002) are other risk factors for failure. By analyzing the interactions between the type of surgery and the diagnosis, we found that the failure rate of POAG patients receiving combined surgery was significantly increased (univariate OR: 7.29; *p* = 0.023). The severity of glaucoma defined by MD was a suspicious risk factor (Cox hazard ratio = 1.04), but it was not statistically significant (*p* = 0.14) (29). Schlenker et al. (72) and Gabbay et al. (64) demonstrated that white ethnicity was associated with a lower risk of failure (adjusted HR, 0.49; 95% CI, 0.25–0.96; 95% CI 1.9–12.4, *p* = 0.001), whereas diabetes was associated with an increased risk of failure (adjusted HR, 4.21; 95% CI, 2.10–8.45).

Two studies reported potential factors for the requirement for bleb needling (12, 20). Fea et al. (12) demonstrated a significant correlation between the number of needling procedures and 1-day (*r* = 0.24, *p* = 0.006), 1-week (*r* = 0.27, *p* < 0.001), and 1-month (*r* = 0.32, *p* < 0.0001) postoperative IOP. However, Sng et al. (20) indicated univariate logistic regression analysis showed that age (*p* = 0.43), sex (*p* = 0.32), glaucoma subtype (*p* = 0.66), number of preoperative glaucoma medications (*p* = 0.34), preoperative medicated IOP (*p* = 0.88), and preoperative unmedicated IOP (*p* = 0.76) were not correlated with the requirement for bleb needling.

### Post-operative Interventions and Reoperations

The largest part of postoperative interventions and reoperations is needling of the XEN conjunctival bleb, the rate of which was 38.7% (5–62%). In a two-year follow-up study (54), 62% of patients required a needling procedure. In most cases, a needling was required within the first month postoperatively (25%). Less than half of the needled eyes (42%) required one procedure. One study reported that after bleb needling, IOP decreased from 25.4 to 13.3 mmHg (47.6%) (6). José et al. (21) hold the idea that a small, flat, non-diffuse or large persistent fibrosis are indicators that needling was needed. Intervention in the early stages of the wound healing process may be considered more effective in improving long-term outcomes. Repeated XEN implantation has been reported in a small number of studies (8, 19, 20, 29, 53, 59, 60, 73). Filtering surgery, including trabeculectomy, Bearveldt glaucoma implant and Ahmed glaucoma valve, was another IOP-lowering method following failed XEN implantation surgery. In one study, 40% of patients required secondary glaucoma filtration surgery within 12 months in the Blacks and Afro-Latino population (31), suggesting that the increased reoperation rate may be multifactorial, including but not limited to intraluminal scarring and pigment occlusion. Following failed XEN implantation surgery, other types of laser and surgery have been subsequently reported, including selective laser trabeculoplasty (26), iSTENT (55), Cypass (53), and cyclodestructive procedures (19, 26, 53).

### Complications

The published complications of XEN implantation with an inner diameter of 45 μm were shown in Table 2. The most common complication was transient hypotony (9.59%). Hypotony was defined as IOP <6 mmHg regardless of outcome in most studies (16, 19, 27, 29, 33, 34, 36, 50, 51, 74), and only 3 studies defined IOP as <5 mmHg (20, 37, 45). Most patients who experienced hypotony do not require additional surgical intervention and will be relieved within 1 month. The rate of chronic hypotony was low due to an intrinsic flow-limiting design based on the tube length and internal lumen diameter. In a study that introduced XEN in glaucoma secondary to medically uncontrolled uveitis, the rate of early hypotony was much higher than average, given that half of eyes (51.3%) experienced transient hypotony. However, all IOPs had stabilized by 1 month, and 18.9% of eyes required further intervention. Notably, in two studies focused on the diameters of 63 or 140 μm (7, 26), the incidence of transient hypotony was surprisingly not greater than that of XEN with the diameters of 45 μm (8.16 and 4.69%). Hypotony-related complications consisted of choroidal effusion and maculopathy, which occurred in 1.31 and 0.86% of patients post-XEN implantation respectively.

**Table 2:** Complications reported after XEN implantation (*n* = 4,410).

**Complication**	**Number (%)**
Transient hypotony	423 (9.59)
Hyphema	244 (5.53)
IOP spikes	93 (2.11)
Choroidal effusions	58 (1.31)
Implant occlusion	41 (0.93)
Macular edema	40 (0.91)
Implant malposition	39 (0.88)
Shallow anterior chamber	39 (0.88)
Hypotonous maculopathy	38 (0.86)
Bleb leakage	30 (0.68)
Implant exposure	25 (0.57)
≥2 Snellen lines vison loss lasting >1 month	15 (0.34)
Corneal edema	13 (0.29)
Endophthalmitis	6 (0.15)

The second most common complication was hyphema, which was noted in 5.53% of patients. Most of these patients had blood occupying less than one-third of the AC (grade I hyphema), which resolved spontaneously by the first week after surgery (20). One study reported that the eye developed hyphema requiring AC washout due to vision loss caused by blood blockage (55). Transient IOP spikes ≥30 mmHg occurred in 0.67% (29)−21.54% (51) (2.11% on average) following hyphema.

Common device-related adverse events included implant occlusion (0.93%), implant malposition (0.88%), and implant exposure (0.57%). XEN implants were found to be occluded by iris tissue or blood in some studies and these were successfully treated with argon laser iridoplasty (55) or a second XEN device (29). Cases of stent malposition needed to remove or reimplant of the stent (12). Bleb-related complications comprised bleb leakage (0.68%) and dysesthetic blebs (0.01%) that required sutures (34, 53) or revisions (15, 35, 75).

Late-onset endophthalmitis was one of the serious complications following XEN implantation in 0.15% of patients. A case of endophthalmitis was observed 9 months after XEN implantation and a secondary surgical intervention (deep sclerectomy) (17). Another case of late-onset postoperative endophthalmitis in the fourth month postoperatively was treated with intravitreal injection of antibiotics, although microbial cultures remained negative (18). Filtering bleb leakage is a potential cause of endophthalmitis (53). Aqueous misdirection or malignant glaucoma developed in 2 patients 4–5 days after XEN implantation and vitrectomy was necessary in both cases (12, 18). Although the IOP was stable after intervention, visual acuity was reduced to a poor level (1/10) (12). Most of the studies directly specified that no patient lost >2 lines of vision. Only 13 patients (0.34%) reported permanent best-corrected visual acuity loss of ≥2 Snellen lines (26, 29, 51). Macular changes were the most possible etiology, including macular puckering, significant drusen, macular edema, and hypotony maculopathy. Other causes consisted of retinal detachment (29) and cataracts (26, 51). None of the patients suffered from loss of light perception in any of the published studies.

Other rare complications included macular edema (0.91%), corneal edema (0.29%), retinal detachment (0.01%), and retinal vein occlusion (0.01%). There is no detailed explanation of the possible causes, whether these were of clinical significance and whether further treatment is needed.

### Comparison With Other Surgeries

**Figure 4:**
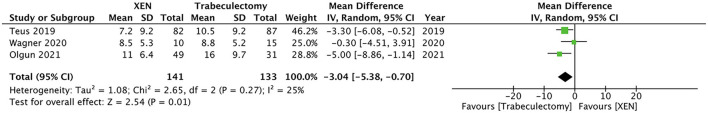
Mean difference (MD) of the reduction in intraocular pressure between the patients after XEN implantation and trabeculectomy.

Eight studies compared XEN surgery with trabeculectomy. Wagner et al. (57) demonstrated that the success rate of trabeculectomy group was similar to the XEN group in the first 6 months. However, the success rate of trabeculectomy was greater than that of the XEN group after 6 months (*p* < 0.05). The reduction in IOP was greater for trabeculectomy compared with XEN (*p* = 0.003). The rate of reoperation for IOP reduction was similar between the two groups (XEN 56.5% vs. trabeculectomy 58.3%), whereas hypotony after surgery occurred more often in the trabeculectomy group (XEN 8.7% vs. trabeculectomy 25.0%). Teus et al. (58) used anterior segment optical coherence tomography (OCT) to compare the morphology of blebs formed when eyes are treated with XEN implants and trabeculectomy. The study showed that the filtering bleb formed after XEN implantation is flatter and smaller with fewer intrableb cystic cavities. Compared with filtering trabeculectomy, the degree of fibrosis of the filtering bleb after XEN implantation is lower. The main reason for a failed trabeculectomy is episcleral or subconjunctival fibrosis (76, 77). The bleb wall was thicker after XEN implantation, which represented more functional blebs. Olgun et al. (78) reported during short-term follow-up, trabeculectomy caused more endothelial cell damage than XEN gel stent implantation. A pooled analysis with a random-effects model showed that IOP decreased more in trabeculectomy group with no significant difference; MD = −3.04 mmHg (95% CI −0.70 to −5.38, *p* = 0.01; Figure 4). Marcos Parra et al. (39) observed that the decrease in IOP in the trabeculectomy group was significantly greater than that in the XEN group (*p* = 0.001), and the reduction in topical glaucoma medications was similar. In terms of success rates, the proportion of patients with IOP ≥6 and ≤ 16 mmHg was slightly lower at 66.2% in the XEN group than 78.6% in the trabeculectomy groups (*p* = 0.1317). The incidence of hyphema and AC flattening was significantly increased compared with that in the trabeculectomy group, whereas the requirement of needlings was much higher in the XEN group. Schlenker et al. (72) demonstrated that the rates of complete success and qualified success for both interventions were similar for the threshold of 6–17 mmHg. Trabeculectomy leads to more transient complications, which are mostly driven by leaks or dehiscences, such as shallow AC. In terms of the reoperation rate, two studies (40, 79) proved that the frequency of necessary postoperative needling procedures was higher in XEN group than in the trabeculectomy group. Basílio et al. (80) evaluated and compared the quality of life of patients after XEN implantation and trabeculectomy through the Glaucoma Symptom Scale (GSS) questionnaire. The results showed that there was no significant difference in quality of life between the two groups.

In three other studies, XEN was compared with other procedures. Compared with gonioscopy-assisted transluminal trabeculotomy (GATT), XEN implantation could achieve more improvement in best-corrected visual acuity and a greater reduction in IOP and the number of antiglaucoma medications (*p* < 0.05) (38). This study revealed that the reduction in IOP was greater in the XEN group with higher medication dependence than in the GATT group, which may be due to the wound healing response in the subconjunctival area. The most common adverse event in both groups was hyphema, and endophthalmitis occurred in the XEN group. Similar to trabeculectomy, both the XEN gel stent and the PRESERFLOTM MicroShunt drain aqueous humor into the subconjunctival space. However, the MicroShunt is typically implanted through the ab-externo approach. Scheres et al. (37) demonstrated that XEN and PRESERFLOTM MicroShunt implantations achieved comparable results in IOP reduction and success rates in POAG eyes. Lower IOP values were found in the MicroShunt group at all time points, but this difference was not statistically significant at the last follow-up. The requirements for bleb needling and additional glaucoma surgery procedures were similar in both groups. In another study comparing XEN implantation and EX-PRESS drainage device implantation (15), EX-PRESS showed superiority in terms of success rate although XEN implantation could reduce the risk of hypotony and choroidal effusion with fewer postoperative clinical visits. In another 9-month follow-up study, non-penetrating deep sclerectomy (NPDS) with MMC was compared with XEN gel stent implantation with MMC (48). The mean reduction in IOP between baseline and the last follow-up was −18.9 ± 5.2% and −25.6 ± 4.3% in the XEN and NPDS groups, respectively (*p* = 0.39). The number of early complications and the number of needling procedures were similar between the two groups. A significant difference of 14.82 min in operation time (XEN 39.09 ± 12.75 min vs. NPDS 52.97 ± 14.37 min, *p* < 0.001) was noted, which made it possible to perform more procedures when XEN was used. In a 3 year follow-up study, Nuzzi et al. (42) evaluate the efficacy of XEN implantation, Cypass, trabeculectomy, and Baerveldt glaucoma implantation. The IOP reduction >20% compared to baseline was 39.1, 55.6, 84.6, and 86.7% respectively. The rate of needling after XEN implantation was the highest, as high as 94.4%.

Eight non-randomized studies compared the outcomes between XEN standalone and XEN combined with cataract surgery. Six studies directly noted that there was no significant difference between the two groups (9, 19, 43, 53, 63, 81). Fea et al. (12) demonstrated that compared with the combined group, more patients in the standalone group achieved complete success with IOP ≤ 14 mmHg and no antiglaucoma medications (41.6 vs. 22.9%, respectively; *p* = 0.03). At 1 week, IOP in the standalone group was significantly reduced compared with that in the combined group (*p* = 0.04), but no significant difference was found in the follow-up. Another study (47) showed that the number of antiglaucoma medications in the standalone group was considerably reduced compared with that in the phaco + XEN group (0.76 vs. 1.4; *p* = 0.06).

## Discussion

This article provides the latest results on the efficacy and safety of ab-interno XEN gel stents. Previous studies have found that this new treatment for various types of glaucoma can reduce IOP by approximately 35%, and the final average value is close to 15 mmHg. In all studies, this reduction was accompanied by a decrease in the number of antiglaucoma medications, approximately 2 classes of medication. The 2-year complete success rate was 21.0–70.8% using the strict criteria originally designed to record the success rate in filtration surgery. The qualified success rate was 34.0–86.0% after 2 years. The largest proportion of reoperation and postoperative interventions was needling of the XEN conjunctival bleb, the rate of which was 38.7% with an excellent IOP-lowering effect (48.7%). Needling should be considered as a part of routine postoperative treatment. Approximately half of the needled eyes required only one procedure. A diagnosis of POAG and requiring needling revisions was postulated as a risk factor for failure. White ethnicity was associated with a lower risk of failure, whereas diabetes was associated with an increased risk of failure. For complications, the most common complication was transient hypotony (9.59%) followed by hyphema (5.53%) and IOP spikes (2.11%). The incidence of vision-threatening complications was very low at <1%.

XEN gel stent is widely implanted through ab-interno approach, however it can be successfully implanted ab-externo as well (59, 62, 75, 82, 83). Vera et al. (84) have verified that both ab-interno and ab-externo approaches for XEN implantation allowed surgeons to better optimize surgery according to the patient's personal conditions, and allows customized surgery to better adapt to the surgeon's preferences. Tan et al. (59), Ucar and Cetinkaya (62) and Do et al. (75) reported that there were no differences in outcomes between ab-interno and ab-externo approaches of the XEN implantation in terms of the IOP reduction and the success rate. Great interest has been expressed in the rate of needling in eyes undergoing XEN implantation. Nuzzi et al. (42) reported the rate of needling after ab-interno XEN implantation was as high as 94.4%. However, many studies (62, 75, 82, 83) have demonstrated that the ab-externo XEN implantation could reduce the rate of needling to as low as 11.8%. They hold a similar view that through ab-external implantation, blunt and broad dissection between Tenon's tissue and scleral could form a better separation between the tissue and the distal end of the gel stent, which helped to reduce the requirement to perform needling postoperatively.

At present, the most frequently performed procedure to combat glaucoma is trabeculectomy (85), relieving the intraocular pressure by draining aqueous to the subconjunctival space and representing the gold standard for surgical treatment of glaucoma. Although it effectively reduces IOP and is cost-effective, it requires close follow-up because of potential complications, such as shallow AC and bleb-related adverse events, which may lead to severe vision loss (86). Similar to trabeculectomy, XEN implantation allows subconjunctival filtration to form a permanent outflow channel to reduce IOP from the AC to the subconjunctival space. The primary advantage of XEN compared with trabeculectomy is that it is a less time-consuming procedure with less surgical trauma, which causes lower rates of intra- and postoperative complications (46). Although the lack of randomization may be unfortunate, there were several interesting studies comparing the safety and efficacy of XEN implantation to trabeculectomy in patients with POAG. The decrease in IOP was greater in patients after trabeculectomy compared with those after XEN implantation. These authors demonstrate that there is no significant difference in the relative risk of failure between XEN implantation and traditional trabeculectomy. Transient hypotony after surgery occurred more frequently in the trabeculectomy group. The rate of hyphema and AC flattening was significantly greater in the trabeculectomy group, whereas the requirement of needling was greater in the XEN group.

The multicentre studies by Kirwan et al. (85) of 428 eyes and the TVT study (87) of 117 eyes confirmed a higher rate of IOP reduction with trabeculectomy of 46.1 and 46.0% over 2 and 3 years of follow-up, respectively, when comparing the IOP reduction of XEN to trabeculectomy. which was much higher than we reported in our review on XEN implantation (35%). With less trauma intraoperatively and the specific designed tube to prevent excessive drainage, the rate of shallow AC after XEN implantation was much lower than that after trabeculectomy (0.88 vs. 0.90–3%). Loss of 2 or more Snellen lines from baseline visual acuity had occurred in 15% of patients and was lower in the stent group than in the trabeculectomy group at 3 years. Regarding bleb needling to lower IOP, the rate after XEN implantation was much higher than that after trabeculectomy (38.7 vs. 16 and 20%). Subconjunctival fibrosis has been considered a key factor leading to surgical failure and postoperative intervention such as needling. Although the degree of conjunctival manipulation in XEN implantation is lower than that in trabeculectomy, postoperative loss of IOP control due to subconjunctival fibrosis is more common. Marcos Parra et al. (39) found the incidence of needling and bleb fibrosis was greater in the XEN implant group. However, in a study using OCT to evaluate the morphology of blebs, Teus et al. (58) found high reflectivity regions in 40% of patients who received trabeculectomy, which was considered a sign of subconjunctival fibrosis, but not as high as in the blebs formed after XEN implantation. Therefore, further studies are required to investigate the incidence and difference in mechanisms between these two procedures.

Both the Ahmed and Baerveldt implants are 2 frequently used aqueous shunts for glaucoma. The Ahmed Versus Baerveldt (AVB) study (88) showed that the reduction in IOP was 47% in the Ahmed group and 57% in the Baerveldt group after 5 years, and these values were considerably greater compared with that found for the stent (35%) in this review. The AVB study reported that the most common complications were shallow AC (15% Ahmed, 17% Baerveldt), choroidal effusions (13% Ahmed, 16% Baerveldt), and persistent corneal edema (11% Ahmed, 12% Baerveldt), all of which were lower with the XEN stent. Bleb needling was necessary in 3% of patients in both aqueous shunt groups and in 38.7% of patients after XEN implantation. Therefore, the efficacy of the XEN gel stent have less reduction in IOP to that of trabeculectomy and other aqueous shunt procedures with fewer complications, but at the price of more needlings.

To date, cost-effectiveness evidence for the XEN gel stent is not available (89), which will be the main consideration that will definitely affect the acceptance of new surgical procedures. Theillac et al. (48) suggested that compared with the traditional filtration surgery, XEN implantation could reduce operation time, which could be used to perform other surgical procedures, and offset the additional cost. Marques et al. (90) and Busch et al. (60) intended to evaluate the learning curve of XEN gel stents with several surgeons from different professional fields. It has been demonstrated that for experienced surgeons and novice residents, XEN implantation showed a fast learning curve. By the time of the sixth implantation, the average operation time and the incidence of complications were reduced in both groups, which was not related to the surgical background or expertise. A shorter learning curve and shorter operation time than other procedures will influence surgeons' choice of diverse microinvasive surgeries.

Different opinions have been raised on whether ethnicity is an influencing factor of the surgical failure rate. Laroche et al. (31) found that among the Black and Afro-Latino patients who received XEN implantation, 40% required additional surgery within 12 months. The success rate was lower than that of studies conducted in a predominantly Caucasian population because the amount of pigment in the iris was significantly higher in the Black and Afro-Latino populations (91), resulting in pigment obstruction of the XEN gel stent. Subconjunctival scarring and fibrosis may be other possible causes of the increased surgical failure rate in Blacks and Afro-Latino patients. Gabbay et al. (50) hold a similar opinion that more postoperative interventions were conducted for the non-Caucasian group. Chao et al. (28) demonstrated that a reoperation rate as high as 45.9% may be related to patients of Chinese ethnicity. Previous studies (92–94) have reported that Asian and Black/African ethnicities exhibit an increased risk of failure with trabeculectomy. Broadway et al. (95) found that the number of fibroblasts and macrophages in the superficial and deep layers of the conjunctival propria in African descent patients was higher than that in European descent patients. This finding partly explains the high tendency of scar formation and failure after filtration surgery in African descent patients. However, the other two studies (27, 51) showed that ethnicity had no statistically significant impact on outcomes. The difference of this may be due to the fact that Asian or Black patients in these two studies accounted for <25%. Therefore, further studies should be performed to investigate the impact of ethnicity on the success and failure rate after XEN implantation.

Although XEN gel implantation is a novel procedure, it has long-term potential in the treatment of glaucoma. Future large-scale, randomized clinical data will help surgeons develop personalized management strategies for each patient. First, it is urgent to study mixed ethnicity or African/Asian populations because most published studies were on Caucasian patients. Second, more studies are required to compare the characteristics of blebs formed when eyes are treated with XEN implants and trabeculetomy or aqueous shunts, which is one of the major factors influencing the outcomes. Third, needling rates and effects should also be studied because minimal conjunctival tissue dissection was required during implantation of the device; however, excessive manipulation of conjunctiva was introduced by the needling procedure. Finally, although the XEN device used in angle-closure glaucoma has been studied, more research and large-scale, standardized, randomized studies are needed to evaluate the outcomes for patients with angle-closure glaucoma. If XEN gel stents are effective in treating such cases, they may become a choice for surgeons in the face of various types of glaucoma.

## Conclusions

The XEN gel stent is the first ab-interno MIGS method used to drain aqueous to subconjunctival space. This implant obtained the CE mark in December 2015 and was approved by the FDA in November 2016. XEN can be expected to reduce IOP by approximately 35%, and the final average value is close to 15 mmHg, which is accompanied by a decrease in the number of antiglaucoma medications, approximately 2 classes of medication. Further studies should be performed to investigate the impact of ethnicity on the success and failure rate after XEN implantation.

## Data Availability Statement

The raw data supporting the conclusions article will be made available by the authors, without undue reservation.

## Author Contributions

X-zC and Z-qL searched literatures, analyzed and interpreted the patient data, and drafted the manuscript regarding the XEN gel stent used for OAG. M-yL, KL, YM, and K-yY helped to search literatures. H-jW designed the work and substantively revised it. All authors have read and approved the final manuscript.

## Funding

This work was supported by the program of development and cultivation of medical innovative varieties and industrial support, Beijing Municipal Science and Technology Commission (Grant No. Z191100007619045) and National Natural Science Foundation of China (Grant No. 61634006). The funders had no role in the study design, data collection and analysis, decision to publish, or preparation of the manuscript.

## Conflict of Interest

The authors declare that the research was conducted in the absence of any commercial or financial relationships that could be construed as a potential conflict of interest.

## Publisher's Note

All claims expressed in this article are solely those of the authors and do not necessarily represent those of their affiliated organizations, or those of the publisher, the editors and the reviewers. Any product that may be evaluated in this article, or claim that may be made by its manufacturer, is not guaranteed or endorsed by the publisher.

## Abbreviations

IOP: intraocular pressure
OAG: open-angle glaucoma
MIGSs: Minimally invasive glaucoma surgeries
AC: anterior chamber
POAG: primary open-angle glaucoma
PACG: primary angle-closure glaucoma
FDA: Food and Drug Administration
RCTs: randomized control trials
PRISMA: Preferred Reporting Items for Systematic Reviews and Meta-Analyses
MD: mean difference
CIs: confidence intervals
MMC: mitomycin C
TVT: Tube vs. Trabeculectomy
OCT: optical coherence tomography
GSS: Glaucoma Symptom Scale
GATT: gonioscopy-assisted transluminal trabeculotomy
NPDS: non-penetrating deep sclerectomy
AVB: Ahmed vs. baerveldt.
